# Change of SPARC expression after chemotherapy in gastric cancer

**DOI:** 10.7497/j.issn.2095-3941.2014.0023

**Published:** 2015-03

**Authors:** Yong-Yin Gao, Ru-Bing Han, Xia Wang, Shao-Hua Ge, Hong-Li Li, Ting Deng, Rui Liu, Ming Bai, Li-Kun Zhou, Xin-Yuan Zhang, Yi Ba, Ding-Zhi Huang

**Affiliations:** Department of Gastrointestinal Medical Oncology, Tianjin Medical University Cancer Institute and Hospital, National Clinical Research Center for Cancer, Key Laboratory of Cancer Prevention and Therapy, Tianjin 300060, China

**Keywords:** Secreted protein acidic and rich in cysteine (SPARC), gastric cancer (GC), immunohistochemistry, chemotherapy

## Abstract

**Objective:**

The expression of tumor biomarkers may change after chemotherapy. However, whether secreted protein acidic and rich in cysteine (SPARC) expression changes after chemotherapy in gastric cancer (GC) is unclear. This study investigated the influence of chemotherapy on SPARC expression in GC.

**Methods:**

Immunohistochemistry was used to analyze SPARC expression in 132 GC cases (including 54 cases with preoperative chemotherapy and 78 cases without preoperative chemotherapy). SPARC expression of postoperative specimens with and without preoperative chemotherapy was assessed to analyze the influence of chemotherapy on SPARC expression.

**Results:**

SPARC was highly expressed in GC compared with the desmoplastic stroma surrounding tumor cells and noncancerous tissues. High SPARC expression was correlated with invasion depth, lymph node, and TNM stage. After chemotherapy, a lower proportion of high SPARC expression was observed in patients with preoperative chemotherapy than in the controls. For 54 patients with preoperative chemotherapy, gross type, histology, depth of invasion, lymph node, TNM stage, and SPARC expression were related to overall survival. Further multivariate analysis showed that lymph node, histology, and SPARC expression after chemotherapy were independent prognostic factors.

**Conclusion:**

SPARC expression may change after chemotherapy in GC. SPARC expression should be reassessed for patients with GC after chemotherapy.

## Introduction

Gastric cancer (GC) is the fourth most common cancer worldwide and the second leading cause of cancer-related death^1^. Chemotherapy efficiency is poor mainly because of its poor chemosensitivity. GC has a low objective response rate (ORR) of 10% to 40% and has median overall survival (mOS) of less than 1 year^1^. ToGA showed that trastuzumab in combination with chemotherapy can further improve the mOS by 2.7 months compared with chemotherapy alone for patients with HER2-positive advanced GC^2^. With regard to high heterogeneity and genetic complexity, the key to further improve treatment efficacy is individualized treatment for patients with different phenotypes. Nonetheless, effective individualized treatment is increasingly dependent on molecular phenotypes and optimal utilization of effective agents.

Nanoparticle albumin-bound paclitaxel (Abraxane; nab-paclitaxel), a novel, biologically interactive form of paclitaxel, significantly improved outcome in pancreatic cancer^3^ and breast cancer^4^. Nab-paclitaxel also showed promising activity and well-tolerated toxicities in previously treated unresectable or recurrent GC^5^, resulting in an ORR of 27.8% and mOS of 9.2 months. Thus, nab-paclitaxel is a new treatment choice. Recent studies revealed that nab-paclitaxel showed promising activity, which might be enhanced by utilizing the binding of albumin to secreted protein, acidic and rich in cysteine (SPARC) and enhanced tumor accumulation of chemotherapeutic agents^5,6^.

SPARC belongs to the matricellular family of proteins^7^ and participates in cell proliferation, migration, adhesion, and angiogenesis in many human malignancies^8^. SPARC has been described as overexpressed in GC tissue and is associated with GC cell invasion and metastasis, thereby providing a prognostic role for GC patients^9^. SPARC is related to the clinical outcome of docetaxel treatment^10^. In addition, studies showed that SPARC expression was associated with the prognosis of patients with pancreatic cancer^11^ and the response of patients with head and neck cancers to nab-paclitaxel^12^. These findings indicate that SPARC is an important phenotype associated with the efficacy of taxanes, especially for nab-paclitaxel. Previous studies on non-small cell lung carcinoma^13^ and breast cancer^14^ showed that tumor phenotypes may change after chemotherapy. However, whether SPARC expression changes after chemotherapy in GC is unclear. Further clarification of the status of the SPARC phenotype under the intervention of chemotherapy will have clinical implications for the reasonable arrangement of chemotherapy and treatment decisions for GC patients.

Therefore, this study examined the effect of chemotherapy on SPARC expression in GC. In addition, the expression and distribution of SPARC and the correlation between SPARC and clinicopathological factors were investigated.

## Materials and methods

### Patients and tissue samples

Between January 2007 and December 2012, 132 patients with GC who underwent gastric resection at Tianjin Medical University Cancer Institute and Hospital were enrolled in this study. These 132 patients included 54 patients with preoperative chemotherapy (group A) and 78 control patients without preoperative chemotherapy (group B). All patients in group A underwent less than six cycles of preoperative chemotherapy, and subsequently, radical resection of GC. The postoperative tumor specimens of group A and group B were selected to analyze the effect of chemotherapy on SPARC expression. Among the group A patients with preoperative chemotherapy, 15 underwent taxanes-based chemotherapy and 39 underwent platinum/fluoropyrimidine-based chemotherapy. All of tumor tissues were diagnosed at the Departments of Gastrointestinal Surgery and Pathology. All of the non-cancerous tissues selected in this study were obtained far from the tumor. This study was conducted with the approval of the Hospital Ethics Committee. All patients signed a written informed consent.

## Immunohistochemistry

Immunohistochemical analysis using the avidin-biotin complex (ABC) method^15^ was performed to study SPARC protein expression. Briefly, slides were baked, deparaffinization with xylene, and rehydrated. The sections were submerged into citrate antigenic retrieval buffer for antigenic retrieval, after which slides were peroxidase blocked in 3% hydrogen peroxide to quench endogenous peroxidase activity. Sections were incubated with 1:1,500 dilution of rabbit polyclonal anti-SPARC (Abcam UK) overnight at 4 °C. Sections of pancreatic cancer specimens were used as positive controls, and the sample incubated with phosphate-buffered saline rather than primary antibody was used as a negative control. After washing, tissue sections were incubated with secondary antibody. Staining was detected using DAB. Then, the specimens were counterstained with hematoxylin, dehydrated, and mounted. The cytoplasm stained with buffy was scored as SPARC positive. Sections were evaluated independently by two observers based on the proportion of positively stained tumor cells and intensity of staining^9^. The tumor cell proportion was scored as follows: 0 (≤5% positive tumor cells), 1 (6% to 25% positive tumor cells), 2 (26% to 50% positive tumor cells), and 3 (≥51% positive tumor cells). Staining intensity was graded according to the following criteria: 0 (no staining), 1 (weak staining, light yellow), 2 (moderate staining, yellow brown), and 3 (strong staining, brown). The staining index was calculated as the product of the staining intensity score and the proportion of positive tumor cells. Using this method of assessment, we evaluated SPARC expression in benign gastric epithelia and malignant lesions by determining the staining index with scores of 0, 1, 2, 3, 4, 6, or 9. The cutoff values for the high and low SPARC expression levels were selected based on the measurement of heterogeneity using the log-rank test with respect to overall survival (OS). The optimal cutoff value was identified as follows: a staining index score ≥4 was used to define tumors with high SPARC expression, and a staining index score ≤3 was used to indicate tumors with low SPARC expression.

### Statistical analysis

All statistical analyses were conducted using SPSS17.0 software. When appropriate, correlation coefficients between protein expression and clinicopathological findings were estimated using the Pearson correlation method, χ^2^ test, or Fisher exact tests. OS was described as the period from the first day after diagnosis to the date of death or last follow-up. Survival curves were estimated using the Kaplan-Meier method, and the log-rank test was used to compute the differences between curves. Cox’s proportional hazards model was used in multivariate analysis to identify the independent predictors of survival. A *P* value <0.05 was considered statistically significant.

## Results

### SPARC expression in gastric cancerous tissues and noncancerous mucosa as demonstrated by immunostaining

**Figure 1:**
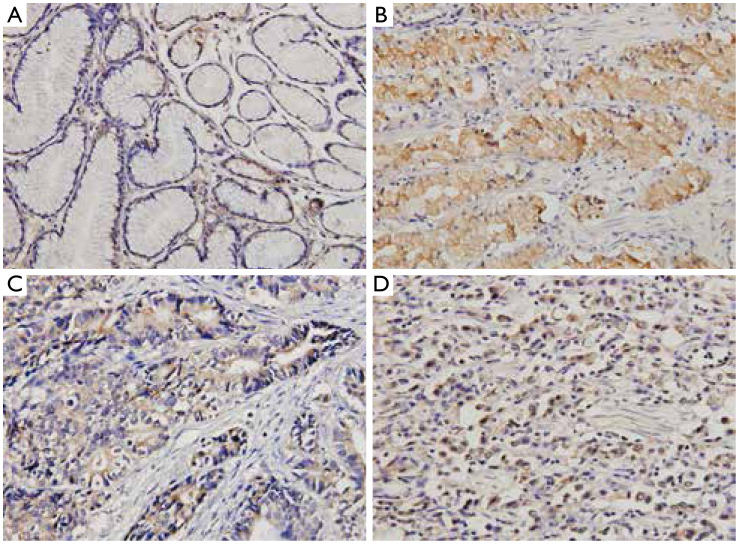
Immunohistochemical staining for SPARC in GC lesions and noncancerous tissues (IHC, ×400). (A) Noncancerous mucosa; (B) GC; (C) intestinal-type GC; (D) diffuse-type gastric cancer. Positive staining of SPARC is indicated by a dark brown color. SPARC expression was stained mainly in the gastric cancer cells and less intensively in the stroma cells. SPARC, secreted protein, acidic and rich in cysteine; GC, gastric cancer.

In normal gastric tissues, SPARC was expressed at low levels in the cytoplasm of 17 of 24 (70.8%) normal mucosal epithelial cells and 16 of 24 stromal cells (66.7%). In GC tissues, high SPARC expression was detected in 55 (70.5%) of 78 tumors (group B). However, immunoreaction was weak or absent in cells of the desmoplastic stroma surrounding cancer cells. SPARC was mainly localized in the cytoplasm of primary cancer (**Figure 1**).

### Correlation of SPARC expression with clinicopathological characteristics

In 78 previously untreated GC patients (group B), higher SPARC expression in postoperative tumor tissue was significantly associated with depth of invasion, lymph node metastasis and TNM stage (*P*<0.05) (**Table 1**). SPARC expression did not correlate with age, gender, tumor location, tumor size, gross type, histological type^16^, and histologic differentiation (*P*>0.05) (**Table 1**).

**Table 1:** Correlation between the expression level of SPARC and clinicopathological factors of patients with GC who underwent gastrectomy

Characteristics	*n* (%) (*n*=78)	SPARC IH, *n* (%)		*P*
		Low (*n*=24)	High (*n*=54)	
Age (years)				0.619
<65	55 (70.5)	16 (29.1)	39 (70.9)	
≥65	23 (29.5)	8 (34.8)	15 (65.2)	
Gender				0.799
Male	57 (73.1)	18 (31.6)	39 (68.4)	
Female	21 (26.9)	6 (28.6)	15 (71.4)	
Location				0.09
Proximal	19 (24.4)	9 (47.4)	10 (52.6)	
Middle	15 (19.2)	2 (13.3)	13 (86.7)	
Distal	44 (56.4)	13 (29.5)	31 (70.5)	
Size (cm)				0.08
<5	34 (43.6)	14 (41.2)	20 (58.8)	
≥5	44 (56.4)	10 (22.7)	34 (77.3)	
Gross type				0.75
Borrmann I/II	72 (92.3)	23 (31.9)	49 (68.1)	
Borrmann III/IV	6 (7.7)	1 (16.7)	5 (83.3)	
Histology				0.967
Intestinal	23 (29.5)	7 (30.4)	16 (69.6)	
Diffuse	55 (70.5)	17 (30.9)	38 (69.1)	
Histologic differentiation				0.967
Well/moderately	23 (29.5)	7 (30.4)	16 (69.6)	
Poorly	55 (70.5)	17 (30.9)	38 (69.1)	
Invasion depth				0.044
T_1/_T_2_	7 (9.0)	5 (71.4)	2 (28.6)	
T_3_/T_4_	71 (91.0)	19 (26.8)	52 (73.2)	
Lymph node metastasis				0.031
Negative	20 (25.6)	10 (50.0)	10 (50.0)	
Positive	58 (74.4)	14 (23.3)	44 (75.9)	
TNM stage				0.021
I/II	22 (28.2)	11 (50.0)	11 (50.0)	
III	56 (71.8)	13 (23.2)	43 (76.8)	

SPARC, secreted protein, acidic and rich in cysteine; GC, gastric cancer.

In 54 GC patients who underwent preoperative chemotherapy (group A), high SPARC expression after chemotherapy correlated with depth of invasion, lymph node metastasis, and TNM stage (*P*<0.05) (**Table 2**). SPARC expression did not correlate with age, gender, tumor location, tumor size, gross type, histological type^16^ and histologic differentiation (*P*>0.05) (**Table 2**).

**Table 2:** Correlation between the expression level of SPARC and clinicopathological factors of GC patients who underwent preoperative chemotherapy

Characteristics	*n* (%) (*n*=54)	SPARC IH, *n* (%)		*P*
		Low (*n*=29)	High (*n*=25)	
Age (years)				0.764
<65	40 (74.1)	21 (52.5)	19 (47.5)	
≥65	14 (25.9)	8 (57.1)	6 (42.9)	
Gender				0.991
Male	41 (75.9)	22 (53.7)	19 (46.3)	
Female	13 (24.1)	7 (53.8)	6 (46.2)	
Location				0.101
Proximal	24 (42.1)	15 (62.5)	9 (37.5)	
Middle	9 (17.6)	2 (22.2)	7 (77.8)	
Distal	21 (38.9)	12 (57.1)	9 (42.9)	
Size (cm)				0.951
<5	24 (44.4)	13 (54.2)	11 (45.8)	
≥5	30 (55.6)	16 (53.3)	14 (46.7)	
Gross type				0.109
Borrmann I/II	42 (77.8)	25 (59.5)	17 (40.5)	
Borrmann III/IV	12 (22.2)	4 (33.3)	8 (66.7)	
Histology				0.565
Intestinal	39 (72.2)	20 (51.3)	19 (48.7)	
Diffuse	15 (27.8)	9 (60.0)	6 (40.0)	
Histologic differentiation				0.609
Well/moderately	17 (31.5)	10 (58.8)	7 (41.2)	
Poorly	37 (68.5)	19 (51.4)	18 (48.6)	
Invasion depth				0.048
T_1_/T_2_	6 (11.1)	6 (100.0)	0 (0.0)	
T_3_/T_4_	48 (88.9)	23 (47.9)	25 (52.1)	
Lymph node metastasis				0.036
Negative	15 (27.8)	12 (80.0)	3 (20.0)	
Positive	39 (72.2)	17 (40.5)	22 (59.5)	
TNM stage				0.01
I/II	17 (31.5)	14 (82.4)	3 (17.6)	
III	37 (68.5)	15 (32.5)	22 (67.5)	

SPARC, secreted protein, acidic and rich in cysteine; GC, gastric cancer.

### The influence of chemotherapy on SPARC expression

**Figure 2:**
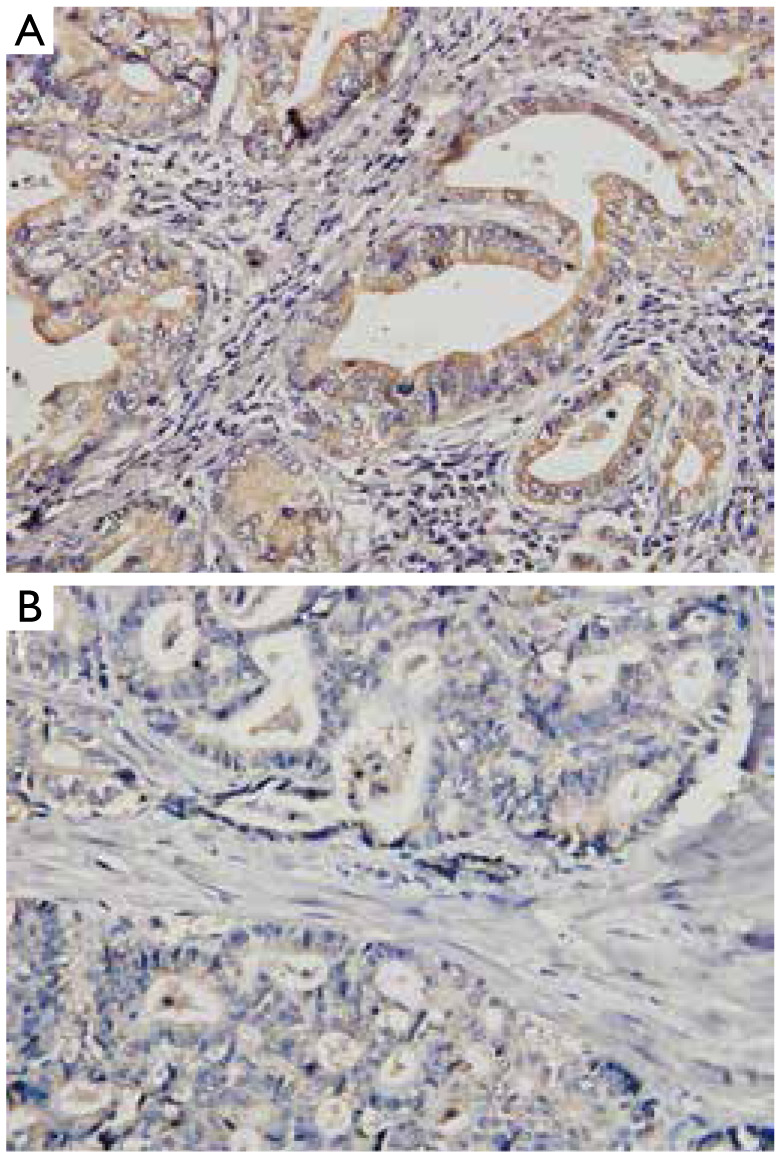
The phenotype of SPARC in relation to chemotherapy (IHC, ×400). (A) High-level SPARC expression in control GC lesions without preoperative chemotherapy; (B) low-level SPARC expression in post-chemotherapy specimens. SPARC, secreted protein, acidic and rich in cysteine; GC, gastric cancer.

We evaluated SPARC expression with and without chemotherapy in specimens from 54 GC patients with preoperative chemotherapy (group A) and 78 GC patients without preoperative chemotherapy (group B). After chemotherapy, a lower proportion of high-level SPARC expression (46.3% *vs*. 70.5%) was observed in group A than in group B (*P*=0.005, by χ^2^ test) (**Figure 2**). Further analysis of preoperative chemotherapy in group A revealed that 15 (27.8%) of 54 GC patients in group A underwent taxanes-based chemotherapy, among which 60.0% showed low SPARC expression in post-chemotherapy specimens. Thirty-nine (72.2%) of 54 GC patients in group A underwent platinum-based chemotherapy, among which 51.2% showed low SPARC expression in post-chemotherapy specimens.

### Correlation between the phenotype of SPARC and patient prognosis

**Figure 3:**
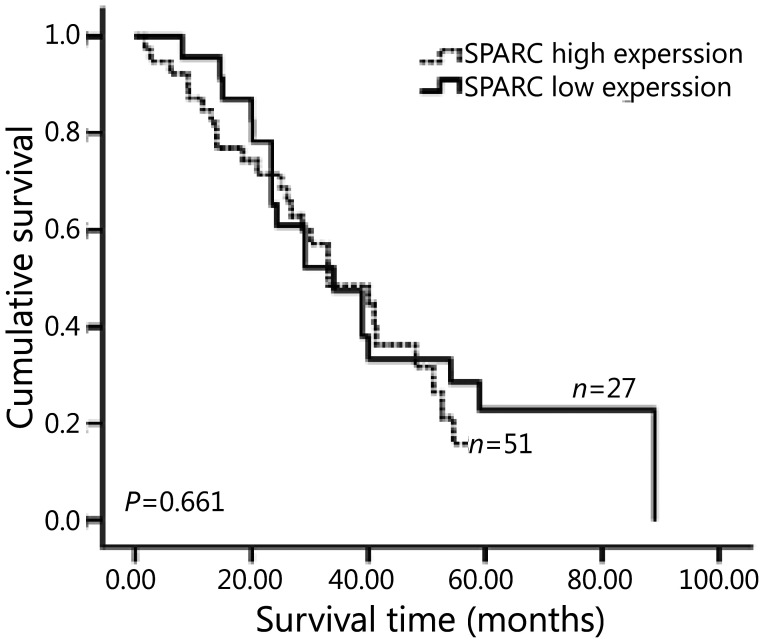
Overall survival of GC patients without preoperative chemotherapy according to the SPARC expression. Kaplan-Meier curves with univariate analyses (log-rank) for 78 GC patients without preoperative chemotherapy stratified as SPARC low expression group and SPARC high expression group; *P*=0.661. SPARC, secreted protein, acidic and rich in cysteine; GC, gastric cancer.

**Figure 4:**
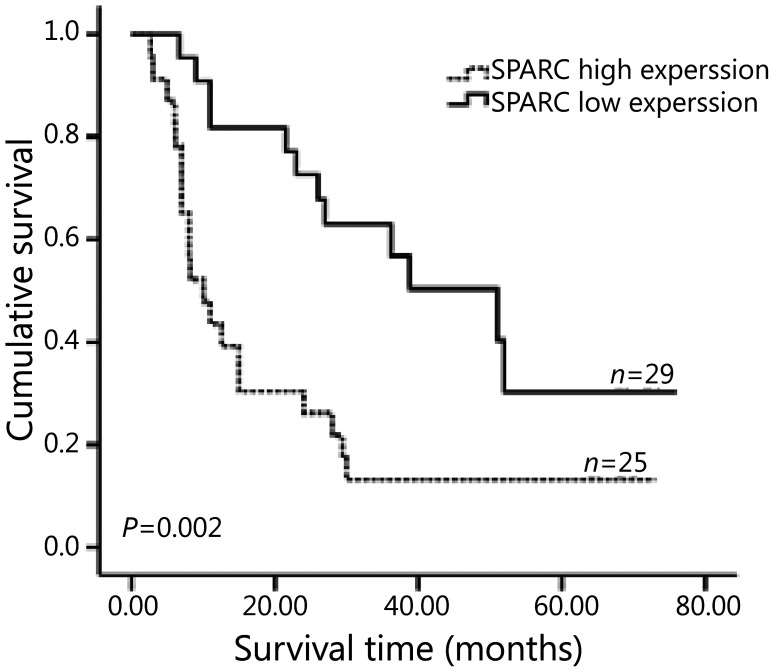
Overall survival of GC patients with preoperative chemotherapy according to the SPARC expression. Kaplan-Meier curves with univariate analyses (log-rank) for 54 GC patients with preoperative chemotherapy stratified as SPARC low expression group and SPARC high expression group; *P*=0.002. SPARC, secreted protein, acidic and rich in cysteine; GC, gastric cancer.

The overall cumulative 2-year survival rate of 132 GC patients (including those in group A and group B) was 64.1% in the low SPARC expression group and 60.6% in the high SPARC expression group (*P*=0.193). Further analysis showed that, in 78 GC patients without preoperative chemotherapy (group B), SPARC expression did not correlate with the prognosis (*P*=0.661) (**Figure 3**). However, in 54 GC patients with preoperative chemotherapy (group A), SPARC expression after chemotherapy correlated with the prognosis. The cumulative 1-, 2-, 3-year survival rates were 81.8%, 72.7%, and 56.7%, respectively, in the group with low SPARC protein expression group; however, these rates were 43.5%, 27.0%, and 13.0%, respectively, in the group with high SPARC protein expression (*P*=0.002) (**Figure 4**).

Univariate analysis showed that other significant prognostic factors for the survival of group A with preoperative chemotherapy included gross type (Borrmann I/II, Borrmann III/IV; log-rank *P*=0.015), histology (intestinal, diffuse; log-rank *P*=0.042), depth of invasion (T_1_/T_2_, T_3_/T_4_; log-rank *P*=0.034), lymph node (N0, N+; log-rank *P*=0.002) and TNM stage (I/II, III; log-rank *P*=0.001) (**Table 3**). Multivariate analysis revealed that lymph node (*P*=0.032), histology (*P*=0.027), and SPARC expression after chemotherapy (*P*=0.024) were independent prognostic factors for the survival of the patients with preoperative chemotherapy (**Table 4**).

**Table 3:** Univariate analysis of prognostic factors of OS for GC patients with preoperative chemotherapy

Characteristics	*n*=54	3-year survival rate (%)	χ^2^	*P*
Age (years)			1.149	0.764
<65	40	39.8		
≥65	14	14.3		
Gender			0.016	0.899
Male	41	32.7		
Female	13	41.5		
Location			0.063	0.969
Proximal	24	29.6		
Middle	9	28.6		
Distal	21	40.3		
Size (cm)			0.989	0.32
<5	24	41.9		
≥5	30	28		
Gross type			5.962	0.015
Borrmann I/II	42	41		
Borrmann III/IV	12	10		
Histology			4.413	0.042
Intestinal	39	46.3		
Diffuse	15	24.3		
Histologic differentiation			3.847	0.05
Well/moderately	17	47.1		
Poorly	37	26.8		
Invasion depth			4.51	0.034
T_1_/T_2_	6	75		
T_3_/T_4_	48	26.2		
Lymph node metastasis			9.591	0.002
Negative	15	68.8		
Positive	39	17.7		
TNM stage			11.018	0.001
I/II	17	70		
III	37	16.7		
SPARC expression			9.552	0.002
Low expression	29	56.7		
High expression	25	13		

GC, gastric cancer; SPARC, secreted protein, acidic and rich in cysteine.

**Table 4:** Multivariate analysis with Cox proportional hazards model for OS of GC patients with preoperative chemotherapy

Variable	HR	95% CI	*P*
Histology	0.378	0.160-0.893	0.027
Lymph node	0.332	0.122-0.909	0.032
SPARC expression after chemotherapy	0.413	0.191-0.891	0.024

GC, gastric cancer; SPARC, secreted protein, acidic and rich in cysteine.

## Discussion

SPARC overexpression was observed in GC and was correlated with invasion, metastasis, apoptosis, and prognosis^9,17,18^. Knowledge concerning SPARC in GC above-mentioned is controversial. We tested SPARC expression in GC tissues and noncancerous tissues and explored its correlation with clinicopathological parameters to further evaluate the correlation of SPARC with the development and progression of GC.

Regarding the cellular origin of SPARC, SPARC may be expressed predominantly in tumor or stromal cells, depending on the type of malignancy^19^. Our immunohistochemical studies showed that SPARC expression was detected mainly in GC cells and slightly in the desmoplastic stroma surrounding tumor cells, normal epithelial cells, and stromal cells. Our results were similar to those of Zhao *et al*.^9^ and Wang *et al*.^19^. However, our results differed from those of other reports demonstrating SPARC staining mainly located in stromal cells^17,20,21^ and that SPARC overexpression in stromal cells surrounding the tumor cells was negatively correlated with clinicopathological factors^17^ and Ki-67 labeling index^21^. Therefore, we hypothesize that SPARC expression in GC cells or stromal cells might play different roles in the carcinogenesis, development, and prognosis of GC. However, further exploration of the molecular mechanism of SPARC in GC is needed to confirm our hypothesis.

In this study, we revealed that SPARC overexpression in GC was associated with depth of invasion, lymph node metastasis, and TNM stage. These results are consistent with the observations of Wang *et al*.^19^ and Zhao *et al*.^9^. A previous study proposed that SPARC may play a key role during the initial steps in the process of tumor invasion and metastasis^22^. SPARC might favor metastatic dissemination of malignant cells through the activation of matrix-degrading enzymes^23^. Moreover, previous studies have shown that a high level of SPARC is often correlated with poor prognosis for patients with GC^9,20^. The results of this study showed that, although in the whole GC patients (group A and group B) SPARC expression did not significantly relate to prognosis, subgroup analysis showed that in group A SPARC expression in post-chemotherapy specimens was associated with poor prognosis, which is probably related to the small size. However, we noted that post-chemotherapy biomarkers were associated with the outcome of patients, a similar finding that could also be observed in breast cancer^24^. The exact mechanism needs to be further explored.

Effective individualized treatment depends on molecular phenotypes, with some evidence of biomarker discordance between pre-chemotherapy and post-chemotherapy tissue specimens^13,14^. In this study, a lower proportion of high-level SPARC expression was observed in specimens with preoperative chemotherapy than in control specimens without preoperative chemotherapy (46.3% *vs*. 70.5%). Therefore, our results suggested that preoperative chemotherapy may alter the phenotype of SPARC. Considering the fact that we cannot reach a definite conclusion based on only a few biopsy specimens, we analyzed the effect of chemotherapy on SPARC expression by comparing SPARC expression between groups A and B, not before and after chemotherapy. A similar method was used in the MAGIC^25^ study. The mechanisms responsible for the discordance may be multifactorial and complicated. Possible explanations may include that chemotherapy may “enrich” to the SPARC-positive GC cells, then kill the SPARC-overexpressioning GC cells or chemotherapy may contribute to the cancer cells inner genetic and phenotypic change in cancer cells.

In addition, further analysis of the preoperative chemotherapy regimens revealed that 60.0% of patients with taxanes-based preoperative chemotherapy showed low SPARC expression in post-chemotherapy specimens. And 51.2% of GC patients with preoperative platinum/fluoropyrimidine-based chemotherapy showed low SPARC expression in post-chemotherapy specimens. Nab-paclitaxel has shown promising activity and well-tolerated toxicities in previously treated unresectable or recurrent GC^5^. Meanwhile, that SPARC binds to the albumin is a remarkable unique mechanism of action^5,6^.

In this study, we proposed that preoperative chemotherapy may alter SPARC expression in previously treated GC patients, particularly in the cohort receiving taxanes-based chemotherapy. These results may provide an important indication that clinicians should consider a re-biopsy of specimens to assess the phenotype of SPARC for previously treated GC patients scheduled for taxanes-based chemotherapy, especially for nab-paclitaxel. However, the limitation of this study is small sample size, which results in conclusions with insufficient power. The findings of this study require further confirmation.
